# Cage aggression in group-housed laboratory male mice: an international data crowdsourcing project

**DOI:** 10.1038/s41598-019-51674-z

**Published:** 2019-10-23

**Authors:** Katie Lidster, Kathryn Owen, William J. Browne, Mark J. Prescott

**Affiliations:** 1 National Centre for the Replacement, Refinement and Reduction of Animals in Research (NC3Rs), Gibbs Building, 215 Euston Road, London, NW1 2BE UK; 2 School of Education, University of Bristol, 35 Berkeley Square, Bristol, BS8 1JA UK

**Keywords:** Animal behaviour, Experimental models of disease

## Abstract

Aggression in group-housed laboratory mice is a serious animal welfare concern. Further understanding of the causes of mouse aggression could have a significant impact on a large number of laboratory animals. The NC3Rs led a crowdsourcing project to collect data on the prevalence and potential triggers of aggression in laboratory mice. The crowdsourcing approach collected data from multiple institutions and is the first time such an approach has been applied to a laboratory animal welfare problem. Technicians observed group-housed, male mice during daily routine cage checks and recorded all incidents of aggression-related injuries. In total, 44 facilities participated in the study and data was collected by 143 animal technicians. A total of 788 incidents of aggression-related injuries were reported across a sample population of 137,580 mice. The mean facility-level prevalence of aggression-related incidents reported across facilities was equivalent to 15 in 1,000 mice. Key factors influencing the prevalence of aggression included strain; number of mice per cage; how mice were selected into a cage; cage cleaning protocols; and transfer of nesting material. Practical recommendations have been provided to minimise aggressive behaviour in group-housed, male mice based upon the results of the study and taking into consideration the current published literature.

## Introduction

Mice are used extensively for the purposes of scientific research, with over 2.7 million mice being used in the UK alone in 2017^1^. Guidelines on their use and care in the laboratory recommend group housing to allow performance of natural behaviours which benefit the welfare of this social species^2–4^. However, natural social groupings for male mice are complex and difficult to directly replicate in the laboratory setting^5^. In the wild, a dominant male will share and defend a territory with several females and their offspring, with the subordinate males dispersing to establish their own territories^6^. In the laboratory, group-housed male mice are confined in cages and have been reported to display dominance hierarchies and associated dominance behaviours^7,8^. Urinary and plantar gland odour cues are important for communication and maintaining these social hierarchies^9^.

Aggression can be defined as the behaviour of one individual that results in a defensive or retaliation response by another. Although aggression is reported in both male and female mice, it is most commonly reported in males. Aggression is part of establishing a dominance hierarchy, but in the confines of the laboratory cage, subordinate animals are unable to escape from aggressors. Aggression in laboratory mice leads to stress, pain and even death^10^. Aggression can also influence the scientific outcomes of experimental studies. For example, pain and/or injury as a result of aggression can lead to changes in physiological parameters, increasing data variability and reducing statistical power and external validity^11^. A common approach to reducing aggression-related injuries is to singly-house the aggressor or injured mouse to prevent further injury. However, singly-housing a social species is itself an additional welfare concern^5^.

Increasing understanding of aggression in group-housed male mice and how to prevent it could have a large welfare impact on a significant number of laboratory animals. Several factors have been identified as contributing to aggression amongst group-housed male mice, including strain^12^, environmental enrichment^13^, group and cage size^14,15^, weaning age^16^ and cage cleaning regime^17^. Studies to investigate aggression in laboratory mice have commonly taken the approach of staged encounters of unfamiliar mice (e.g.^18^). Limited studies have evaluated aggression arising spontaneously in the laboratory setting. Such studies may identify aspects of routine housing and husbandry that could influence aggressive behaviour.

The aim of the current study was to collect data on incidents of aggression to determine the prevalence and triggers of cage aggression in group-housed male mice and to provide an evidence base to inform and support best practice to minimise aggressive behaviour and any single housing of laboratory mice. The study used a crowdsourcing approach (“*the collective wisdom of the crowd to achieve a solution to a problem that effects the crowd*”^19^) and data were collected by animal technicians across participating research establishments. The benefit of using crowdsourcing was the ability to collect data from a very large population of laboratory mice across multiple facilities. This approach also avoids the unnecessary use of experimental animals, in line with the principles of the 3Rs.

## Results

### Prevalence of aggression

The prevalence was calculated as the number of mice (or number of cages of mice) presenting with aggression-related injuries as a fraction of the total number of mice (or total number of cages of mice) in the facility for the period of data collection (see Methods for data collection). Using data provided from all facilities, a ‘snapshot’ of prevalence of aggression was calculated. Data was excluded for four facilities: three due to lack of information on the total number of mice held during the data collection period; and one which used an unconventional mouse strain derived from a wild mouse population (i.e. not the population of interest) which reported an abnormally high incidence of aggression (48%) (n = 1).

**Figure 1:**
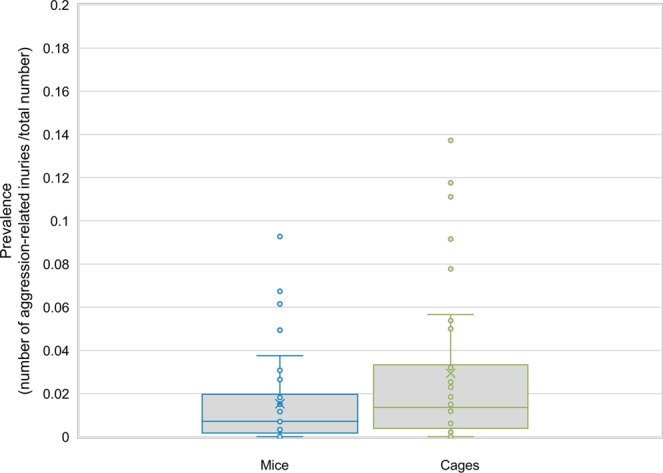
Prevalence of aggression-related injuries in mice (blue) and cages (green) expressed as a proportion (n = 40 facilities). Box plot shows mean, median, upper and lower quartiles. Data falling outside of the upper and lower quartiles range are plotted as outliers.

This left a total of 137,580 mice and 45,412 cages across 40 facilities which were included in prevalence analysis. A total of six facilities reported no incidents of aggression. The size of facilities participating in the study ranged from 80 to 27,206 mice and 18 to 11,965 cages held during the collection period. The mean prevalence of aggression-related injuries was calculated from the mean prevalence from each facility and calculated to be 0.0153 (mice with aggression-related injuries/total number of mice) which equates to 1.53% or ~15 in 1,000 mice. The mean prevalence for cages was calculated to be 0.0294 (cages of mice with aggression-related injuries/total number of cages) which equates to 2.94% or ~29 in 1,000 cages (average of facility means for n = 40 facilities) (Fig. 1). The prevalence data was also calculated for UK institutes only (n = 32) and found to be 0.0147 (mice with aggression-related injuries/total number of mice) which equates to 1.47% or ~15 in 1,000 mice and 0.0223 (cages of mice with aggression-related injuries/total number of cages) which equates to 2.23% or ~22 in 1,000 cages. The difference in prevalence between mice and cages reflects the spread of aggression across different cages.

**Figure 2:**
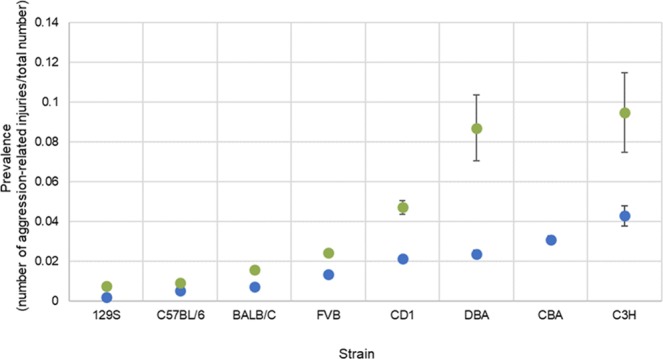
Prevalence of aggression-related injuries in mice (blue) and cages (green) for the top ten most commonly reported strains in the study with 95% confidence intervals. Strains include 129S (n = 5,113 mice, n = 1,307 cages), C57BL/6 (n = 78,487 mice, n = 26,613 cages), BALB/c (n = 6,292 mice, n = 697 cages), FVB (n = 4,388 mice, n = 114 cages), CD1 (n = 2,443 mice, n = 680 cages), DBA (n = 509 mice, n = 92 cages), CBA (n = 937 mice, no data available for cages), C3H (n = 257 mice, n = 74 cages). See Supplementary Table 2 for further information.

The prevalence of aggression varied across different strains. Due to the large variation of strains housed in the facilities during the data collection period, the most commonly reported strains were categorised according to their common strain lineage (i.e. 129S, C3H, C57BL/6, CBA, CD1, BALB/c, DBA, FVB) and prevalence of aggression was calculated (Fig. 2). In total, these categorised strains represent 72% (98,426 of 137,580) of mice observed during the study. Of these strains, strains showing high-prevalence of aggression include C3H, CBA and CD1; strains showing low-prevalence of aggression include 129S, C57BL/6 and BALB/c. A Chi-square test of independence was used to compare the prevalence of aggression-related injuries and showed significant differences between strains when looking at incidents at the mouse (χ2(7) = 318.72, P < 0.001) and cage level (χ2(6) = 202.12, P < 0.001).

### Standard conditions

**Figure 3:**
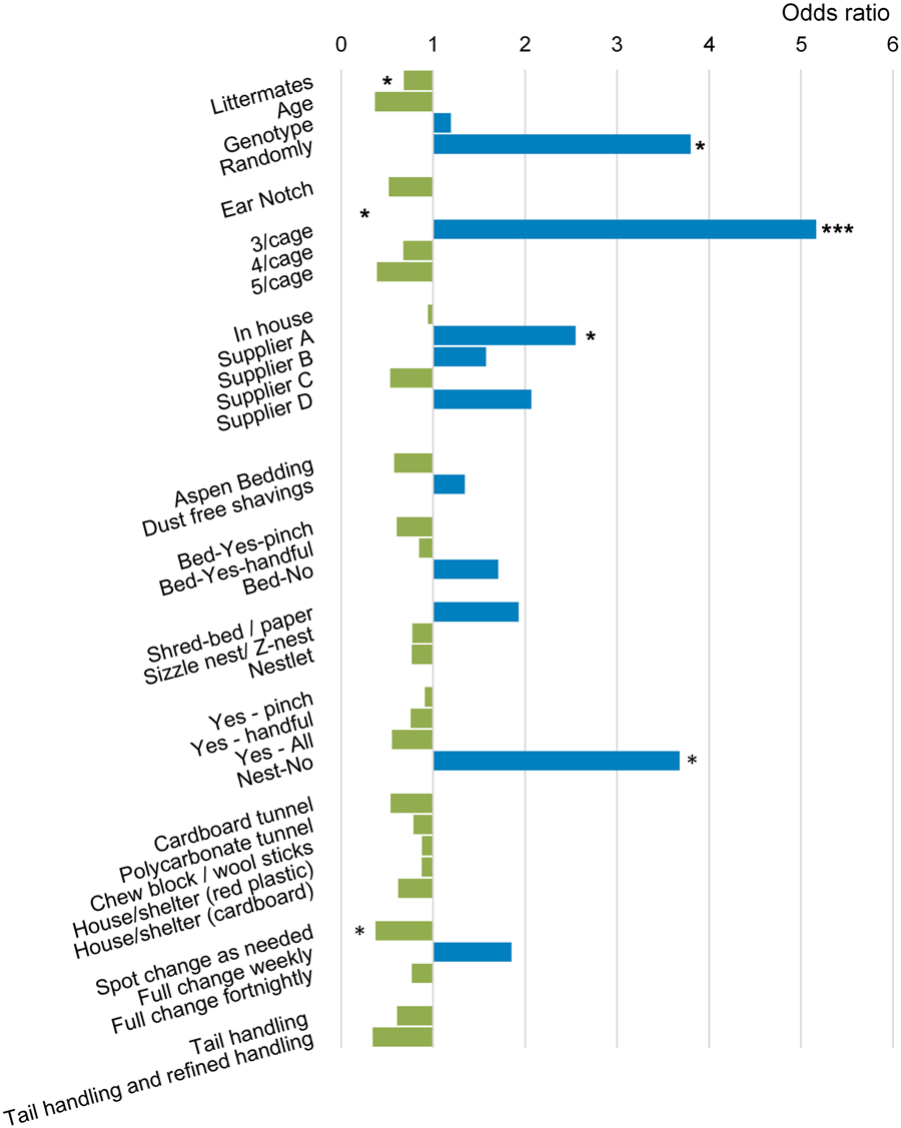
Effects of standard conditions on the prevalence of aggression across facilities (n = 40 facilities). An odds ratio above one (blue) indicates that aggression is more likely when the standard condition variable is present. An odds ratio below one (green) indicates that aggression is less likely when the standard condition variable is present. (***p < 0.001, *p < 0.01). See Supplementary Table 3 for further information.

A summary of the standard conditions can be found in the Supplementary Table 3. The data from each facility was compared with the prevalence of aggression in the facility. The aggregated data from across 40 facilities was combined to identify standard condition variables of interest. The odds ratios of each standard condition being associated with an increase/decrease in aggression are shown in Fig. 3.

Analysis of the data showed that the following standard conditions were associated with a decrease in aggression-related injuries: selecting animals into a cage by littermates (OR = 0.367, P = 0.014), and spot cleaning as needed (OR = 0.374, P = 0.014). The following standard conditions were associated with an increase in aggression-related injuries: selecting animals into a cage randomly (OR = 3.800, P = 0.021), sourcing animals from supplier A (OR = 2.550, P = 0.033), and not transferring nesting material (OR = 3.680, P = 0.039). The number of mice housed per cage also had an impact, with five mice per cage reducing incidents (OR = 0.389, P = 0.014) and three mice per cage increasing incidents (OR = 5.166, P < 0.001).

### Injury log

**Figure 4:**
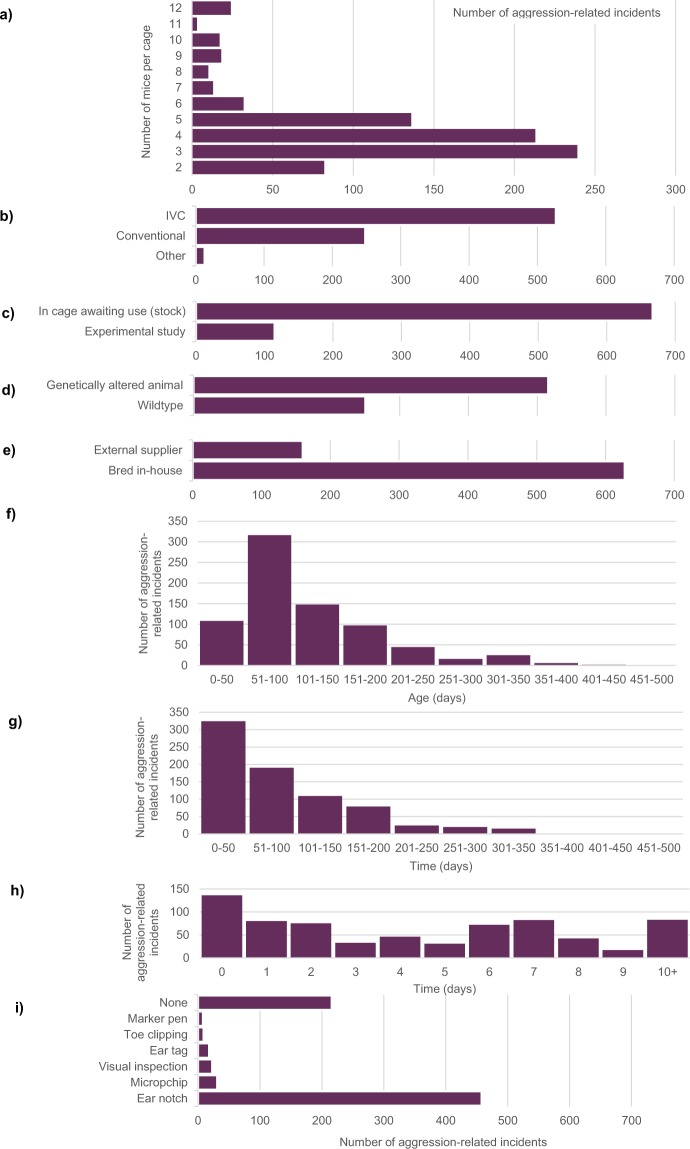
Summary of housing and husbandry conditions for mice showing aggression-related injuries (n = 788 incidents of aggression) for: (**a**) number of mice per cage, (**b**) cage type, (**c**) status of animals, (**d**) genetic status, (**e**) source of animals, (**f**) age of animals, (**g**) time housed with mice in current cage, (**h**) days since last cage change, (**i**) method of identification.

878 incidents of aggression-related injury were reported in total from all 44 facilities that participated. Ninety incidents were excluded due to lack of evidence of the injuries having occurred during the data collection period (e.g., old bite marks, no evidence of behavioural changes) or because the animals were breeding stock. In total, 788 mice were included in the analysis. A descriptive summary of the mice presenting with aggression-related injuries and their housing and husbandry conditions is presented in Fig. 4, showing data on the number of mice per cage, cage type, status of animals, genetic status, source of animals, age of animals, time housed with mice in current cage, days since last cage change and method of identification. Note for the results that follow these are descriptive: we only have the number of injuries observed for each category and not the total number of mice in each category, therefore the observed effects may reflect the high incidence of mice in this category from the total population.

Aggression-related injuries were reported in cages varying from two to 12 mice per cage; the most commonly reported group composition where aggression was observed was three per cage (Fig. 4a). Only six facilities reported holding three mice per cage as a standard condition (see Supplementary Table 3); however, an additional 18 facilities reported aggression-related incidents from mice housed as three mice per cage. This could be due to facilities housing mice at larger stocking densities initially and reducing this to three per cage as mice are removed for mating, experiments or due to aggression.

Most incidents of mouse aggression were observed in mice held in IVC cages (67%, 527 of 788 incidents) compared to conventional cages (31%, 248 of 788 incidents) (Fig. 4b). The majority of mice were held in cages awaiting use (i.e. stock animals) (85%, 667 of 788 incidents) rather than in an experimental study (14%, 115 of 788 incidents) (Fig. 4c). Of those mice that were in an experimental study, a wide range of procedures were reported (e.g. intravenous administration, blood collection, behavioural tests).

**Figure 5:**
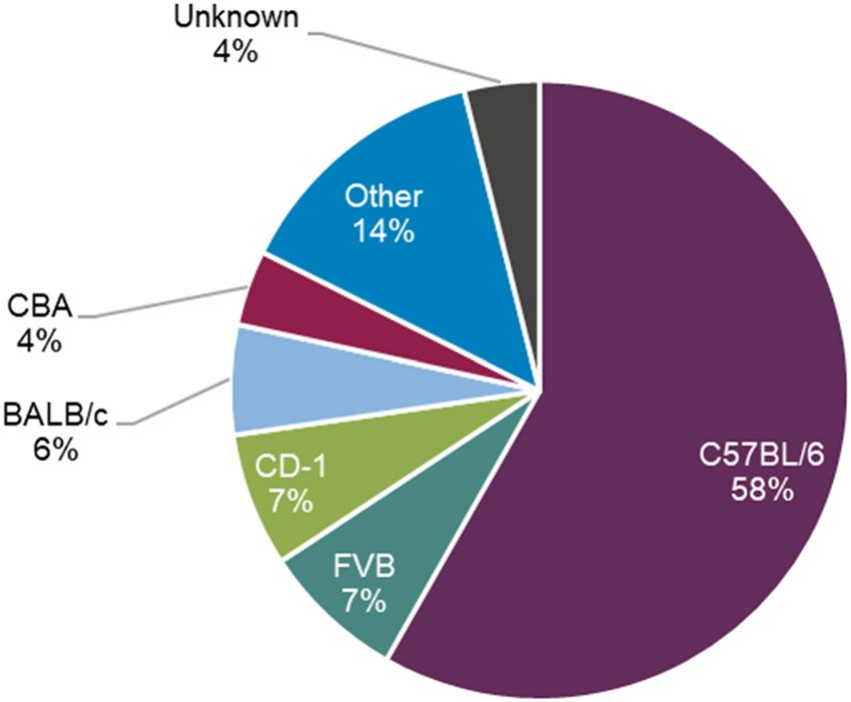
Strains of mice presenting aggression-related injuries (n = 788 mice).

Most incidents of aggression were recorded in mice of C57BL/6 lineage (58%, 459 of 788 incidents) (Fig. 5), which simply reflects the high number of this lineage held during the data collection period and is not a direct reflection of the prevalence of aggression in this strain, which is low (see Fig. 2 for information about prevalence in different strains). Nearly all incidents (97%, 633 of 650 incidents) involved mice with no marked difference in size between other mice in the cage (observations only).

**Figure 6:**
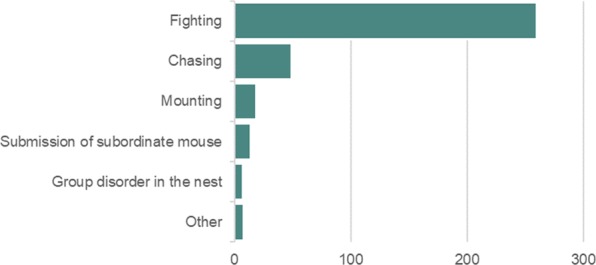
Behavioural changes observed in mice with aggression-related injuries (n = 301 mice).

**Figure 7:**
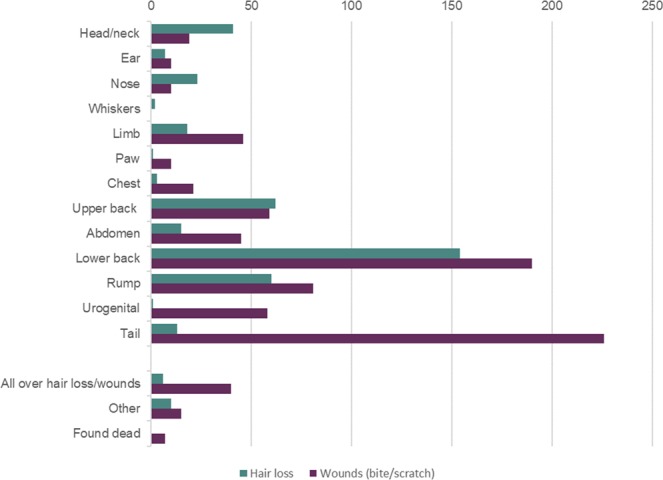
Description of aggression-related injuries, including hair loss (green) and bite or scratch wounds (purple) (n = 788 mice).

A range of behavioural changes were observed in 38% of cases (301 of 788 incidents) (Fig. 6) and were interpreted and classified into categories by each individual facility. Behavioural changes included fighting (33%, 260 incidents), chasing (6%, 49 incidents), mounting (2%, 19 incidents), and submission of a subordinate mouse (2%, 14 incidents). No behavioural changes were observed or reported in the remaining 62% of cases (487 of 788 incidents). Participants provided a description of the aggression-related injuries including wounds and hair loss (Fig. 7). Most wounds were found on the tail (27%, 226 incidents), lower back (23%, 190 incidents) and rump (10%, 81 incidents). Hair loss was mostly reported on the lower back (37%, 154 incidents), upper back (15%, 62 incidents) and rump (14%, 60 incidents).

### Actions to prevent further injury

**Figure 8:**
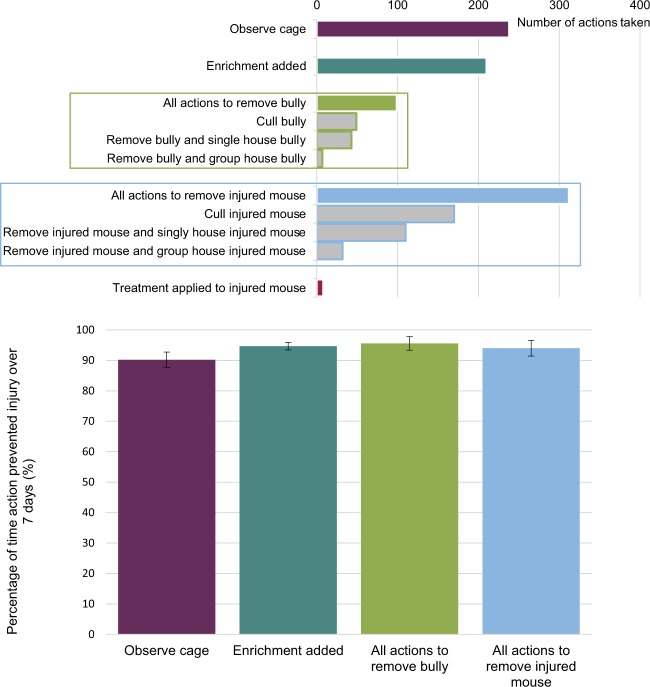
Seven-day follow up period: (**a**) actions taken by participants to prevent further injury, and (**b**) percentage of time the action taken prevented further injury over the seven-day follow-up period (n = 494 incidents of aggression-related injury).

Following observations of aggression-related injuries, participants were asked to report actions taken to minimise further injury (Fig. 8a). This included actions to remove the injured mouse (40%, 312 of 788 incidents) or to remove the aggressor (13%, 99 of 788 incidents), increased observations of the cage (30%, 238 of 788), adding further enrichment (27%, 210 of 788 incidents) and alternatively, treatment applied to the injured mouse (1%, 8 of 788 incidents). More participants removed the injured mouse than removed the aggressor, possibly reflecting the relative ease of identifying the injured mouse in a cage and difficulty in identifying the bully. Other actions reported included singly housing all mice from the cage, changing the bedding material or moving mice to a larger cage. In over 99% (779 of 788 incidents) of reported cases of injuries, action was taken to prevent further injury.

To further investigate the impact of the actions taken to prevent aggression, the cage of animals was observed over a seven-day follow-up period. Data was submitted for the seven-day follow-up period for 494 incidents (Fig. 8b). In over 90% of cases, the action taken prevented further injury (i.e. no further incidents of aggression were recorded). No further incidents of aggression were observed in most cases after action was taken to either add enrichment, remove the bully or remove the injured mouse.

## Discussion

### Prevalence

Prevalence measures the proportion of mice in a population with aggression-related injuries at a certain point of time. We understand this is the first attempt at understanding prevalence of aggression across a very large sample of laboratory mice (137,580 animals) in an observational setting across multiple institutions. The prevalence of male mouse aggression provides a background rate (15 in 1,000 mice), which can be used by other facilities as a benchmark for good practice.

### Strain differences

Strain-specific differences in aggression were observed and provide a snapshot of strains with a higher and lower prevalence of aggression. A comparison of multiple strains together has not previously been reported on this scale, although it is known that there is a strain difference in the levels of aggression^20^. Our study results are generally supported by the existing evidence base, which shows strains with a higher level of aggression include CBA^21^ and CD-1^12,22^ compared to strains with a lower level of aggression such as C57BL/6^23^. In a survey of mainly UK stakeholders^5^, C57BL/6 mice were among the strains most commonly considered “too aggressive to be group housed”. However, this finding may be due to the greater number of C57BL/6 mice held in UK laboratories in comparison to other strains. Our study in fact showed C57BL/6 are amongst the strains showing low levels of aggression. Differences in prevalence of aggression in strains may reflect differences in underlying neural mechanisms controlling behaviour^21^. Subtle genetic alterations can have profound effects on behavioural responses^24^; for example, differences in social behaviours between C57BL/6 and BALB/c mice^25^.

### Standard conditions influencing levels of aggression

The following housing and husbandry variables were found to influence levels of aggression and are discussed in relation to the current literature. Our findings are not strain-specific (i.e. all strains were included in the analysis of standard conditions), which therefore does not take into account the potential variation in environment-interactions across different strains. Much of the previous literature has been conducted in specific strains of interest; these are noted where possible throughout the discussion.

#### Cage cleaning

It was clear from our study that there is variation in cage cleaning regimes with different cage cleaning approaches and frequencies. We found a spot clean when needed was associated with a significantly lower prevalence of aggression in comparison to weekly or fortnightly full cage change. Several studies have shown the effect of cage cleaning on levels of aggression in mice^13,26^. Cage cleaning disturbs scent marks, which temporarily disrupts the social hierarchy of animals in the cage and decreases social stability in the group^27^.

#### Transfer of cage bedding and nesting material

Our study showed 50% of facilities (22 of 44) transferred bedding material (i.e. cage floor substrate) and 86% (38 of 44) transferred nesting material. We found that not transferring nesting material from the soiled cage to the clean cage was associated with an increase in aggression-related injuries. This finding suggests transfer of nesting material helps to reduce aggression.

In efforts to reduce the impact of cage cleaning on aggression, the transfer of nesting material has been previously proposed as good practice^17^. However, both scientific and anecdotal evidence is contradictory on whether transfer of olfactory cues in bedding and nesting material can increase or decrease aggression. For example, Gray and Hurst (1995) recommend mice are transferred to a completely clean cage if aggression is a concern, as they showed partial cleaning elicited aggression in the outbred CFLP strain. Van Loo *et al*. (2000) showed transfer of nesting material resulted in lower levels of aggression in the BALB/c strain. A later study clarified the type of material transferred impacts the level of aggression: transferring nesting material decreases aggression, whereas transferring soiled bedding material increases the level of aggression^28^. This was hypothesised to be due to the bedding material being contaminated with urine containing aggression-eliciting odour cues. The nesting material is kept clear of urine and faeces and contain hormones from glands in the body (e.g. plantar glands) that have been shown to inhibit aggression^17^.

Previous studies have compared type of bedding and its effects on levels of aggression (e.g. corncob bedding contains oestrogen disrupters that increase aggression^29^). Our study did not show any significant differences in aggression due to bedding type; the majority of facilities used aspen bedding (59%, 26 of 44) and only four facilities reported using corn cob bedding.

#### Size of group

Our study found the standard number of mice housed per cage can affect the level of aggression. An increase in the prevalence of aggression was found in facilities which housed mice in cages of three as their standard condition. Previous evidence suggests housing mice in smaller social groups, such as three to five animals per cage, helps to avoid aggression^12,15,30,31^. The different results seen here could be due to the different study approaches and strains studied, which may not be directly comparable. For example, Butler (1980) observed wildtype mice in semi-natural enclosures and found that increasing population number increased the rate of agonistic interactions. Van Loo *et al*. (2001) studied BALB/c mice in laboratory setting, which were randomly allocated to groups of three, five or eight and monitored agonistic behaviour. The authors concluded that dominance hierarchy is more stable in smaller groups and later made a recommendation to house mice in groups of three. Further evidence is required to support this recommendation to house mice in small groups of three given the results presented here. The effect of space allocation on the well-being has recently been reviewed^32^. The group behaviour and social interactions could be due to various environmental factors (e.g. cage size and environmental enrichment) in addition to group size^33^.

#### Method of selection

How mice were selected into the cage was found to be associated with the level of aggression. Selecting cage mates randomly was associated with an increased prevalence of aggression whilst selecting cage mates from littermates was associated with a decreased prevalence of aggression. These results are in accordance with previous literature that highlights the importance of maintaining stable social groups. During randomisation, established social hierarchies are disrupted; aggression then occurs as part of the process to re-establish a new dominance hierarchy. Weber *et al*. (2017) recommended keeping littermates together after weaning to help decrease aggression^10^. Bartolomucci *et al*. (2002) concluded that keeping laboratory mice in same-sex sibling groups from birth provides the ideal social environment for their welfare^34^.

#### Supplier

The source of mice was shown to be associated with the prevalence of aggression at the facility level. Facilities that sourced mice from Supplier A had a greater prevalence of aggression. It should be noted, however, that many facilities used several suppliers and the source of each individual mouse was not linked directly to a given supplier. The suppliers’ identities have been anonymised in this study. More specific details on the methods of shipment (e.g. stocking density) and distance travelled are not available.

### Standard conditions with no effect on aggression

Standard conditions that did not show a significant relationship with the prevalence of aggression in our study include identification method, routine handling method, type of bedding and nesting material. Further discussion has been provided below about the cage environment, temperature and age of weaning, which have been linked to aggression in previous studies.

#### Cage environment

Our study did not find any conclusive results for the effect of environmental enrichment on aggression. The current literature presents mixed conclusions (reviewed by Olsson *et al*.^35^), dependent on strain, enrichment type and availability. Some studies have found mice monopolize and defend structural enrichment such as cage furniture leading to a higher incidence of aggressive behaviour, whereas manipulable enrichment such as nesting material can decrease the incidence of aggression; other studies have found opposite effects^36–38^. Cage complexity has also been shown to have an effect on aggression; partial cage dividers resembling burrows have been shown to reduce aggressive behaviour in 8 week old male BALB/c mice^39^.

#### Temperature

Previous studies have shown increased aggression in the laboratory as ambient temperatures increase from 20 to 25 °C^14^. In our study, participants reported their standard laboratory temperature range, but we were unable to assess the impact of temperature on aggression due to all facilities reporting similar temperature ranges.

#### Age of weaning

Previous studies have shown the potential influence of weaning and early life experience on aggression^40^. The age of weaning was provided by the participating facilities but the lack of variability in the data (37 of 40 facilities reported weaning mice on day 21) meant it was not possible to examine if age of weaning had any effect on aggression.

### Aggression-related injuries and actions taken

The descriptions of injuries in the injury log provide an insight into the most common areas of the body injured following aggression. Injuries typically present on the tail, lower back and rump area, indicating an attack, which is in line with previous reports observed in resident/intruder experiments describing wounds on the dorsal area of mice^41,42^.

A range of actions were taken following observation of an injury. This possibly reflects the lack of guidance in this area. The data collected provides an insight into the range of actions taken to minimise further injury and will be used to inform future NC3Rs studies in this area.

### Study limitations

This study adopted a novel approach using crowdsourcing to achieve a large data set. A key challenge of running a crowdsourcing project is maintaining data quality. There is the potential for inconsistency in the data submitted due to the large number of participants and different approaches for collecting data. To minimise this, we provided learning material and quality checked the submitted data to ensure comprehensive and correct entry of fields in the spreadsheet. Where data was absent or unclear, queries were sent to study participants to ensure the final data set was as complete and accurate as possible.

Participating facilities were predominantly UK academic institutions and may not be fully representative of the broader laboratory animal research community. This was a convenience sample population and participation in the study was voluntary, so responses may be biased towards facilities with lower levels of aggression. However, we consider the responses to be sufficiently representative to draw valid conclusions. Response bias is a limitation of every survey-based research project and might have influenced our findings, but there is no other practical way to achieve information on a large scale. The standard conditions reported by each facility were assumed to be uniform across the facility, however some variability was observed between the standard conditions and the conditions reported for each individual mouse in the injury log report. For example, 18 facilities did not report housing mice three per cage as a standard condition, however they reported incidents of aggression from mice housed in cages with three mice per cage.

The appearance of wounds may not be the best indicator of aggressive behaviour. Mild aggression which did not result in visible wounds may have been missed from the observational study. As a result, the reported prevalence of aggression may underestimate the true prevalence of aggression. Different methods have been developed to evaluate aggression with high sensitivity and specificity, such as the “Pelt Aggression Lesion Scale” which scores subcuticular signs of aggression observed during necropsy^43^. However, our study was limited to observational recordings during daily checks to avoid culling of animals. Further insight into the aggressive behaviour of animals could be studied using automated home cage monitoring systems^44^.

Incidents of injury recorded by participants may have erroneously included animals with ulcerative dermatitis. Ulcerative dermatitis is a common, spontaneous condition in mice resulting in ulcerations on the dorsal cervical and thoracic regions^45^. The recording of injuries associated with aggression was subjective, however, participants were provided with guidance and images on how to identify injuries resulting from fighting. The locations of the injuries reported (i.e. lower back, rump and tail) would suggest that they are related to aggressive attack rather than ulcerative dermatitis, which is usually concentrated in the cervical and thoracic regions.

### Recommendations and future studies

In summary, it is clear from the results that there are many factors that are associated with aggression in group-housed male mice and that aggression is a common problem. Taking steps to prevent aggression in the laboratory would have a significant welfare benefit. Some of the factors that were found to be associated with aggression can easily be managed and we provide recommendations based upon the findings of the study and the current evidence base. Many of these recommendations are small, practical husbandry changes and should not require significant resources to implement.

1. **Consider the strain of mice for your experiment:** If a choice of strain is possible for the scientific aims of the study, consider whether a strain showing a low prevalence of aggression can be used. Bear in mind that some strains (i.e. C57BL/6 mice) may anecdotally be classed as highly aggressive but in fact show low levels of aggression.
2. **Cages should be spot cleaned as needed:** The frequency of cage cleaning should be kept to a minimum to avoid disruption of the olfactory cues in the cage environment. Spot cleaning dirty bedding as needed can keep disturbance to a minimum.
3. **Nesting material should be transferred during cage cleaning:** Transfer of some nesting material from soiled to clean cage helps to stabilise the olfactory cues in a group of mice. The nesting material transferred should be clean and dry. Do not transfer soiled bedding.
4. **Mice should be group-housed with littermates**, **where possible:** Avoid mixing unfamiliar mice together in a cage and instead establish stable groups with littermates, wherever possible. If randomisation is required as part of the allocation process for experimental design, this can be done without mixing unfamiliar mice in a cage. For example, randomisation to treatments can be done by marking individual mice (e.g. by tail markings) and allocating each mouse to a treatment group but allowing cages of littermates to be maintained for housing purposes. In this scenario, the cage would be considered a blocking factor to ensure an even distribution of treatments across the cages. Further information to help researchers with the allocation process can be found on the NC3Rs Experimental Design Assistant (https://eda.nc3rs.org.uk/experimental-design-allocation).
5. **Discuss how mice are shipped from suppliers:** If mice are sourced from external suppliers, discuss the steps suppliers could take to reduce aggression. For example, request that animals are transported with littermates during the ordering process.

In some areas, our study findings were not in accordance with the current published literature and/or were not sufficiently conclusive to allow a recommendation to be made. Additional research in these areas could help further minimise the prevalence of aggression. The NC3Rs provides research funding schemes (www.nc3rs.org.uk/funding), which may help facilitate such projects in the UK.

- **Number of mice per cage**: Our study found that housing mice in three animals per cage was associated with an increased prevalence of aggression. However, published studies (e.g. Van Loo *et al*. 2001) recommend housing mice in small groups. Other factors ought to be taken into consideration, such as, how the mice are selected to be housed together or how a cage is reduced from a higher stocking density.
- **Environmental enrichment:** It is not clear how environmental enrichment influences aggression as empirical evidence is conflicting. Depending on the evidence available, a systematic review and meta-analysis of the literature could provide evidence to support a recommendation (for example, SyRF is a free-to-use online resource for researchers to aid systematic review and meta-analysis of *in vivo* studies: http://syrf.org.uk).
- **Actions taken to minimise further aggression:** The variety of actions taken suggests there is a lack of consensus on good practice. It is preferable to avoid single-housing and to maintain mice in groups where possible. One possibility to avoid isolating the presumed aggressor could be to split groups of mice experiencing aggression into two smaller subgroups, which has been supported by recent research^46^. A controlled study investigating different actions that could be taken would help provide further insight and guidance for minimising further aggression. However, we believe the focus should be on prevention of aggression in the first instance using the recommendations described above.

We intend to use the results of this study to develop hypotheses to test in a second phase study using a similar crowd-souring approach (updates will be made available at www.nc3rs.org.uk/laboratory-mouse-aggression-study).

## Methods

### Data collection

The study was open to all licensed facilities with group-housed male mice. Participation was encouraged via the NC3Rs website (www.nc3rs.org.uk), newsletter, social media accounts (Twitter, Facebook, LinkedIn) and staff contacts; the Institute of Animal Technology (IAT) website (www.iat.org.uk) and newsletter; and online forums and email lists for named persons working under the Animals (Scientific Procedures) Act 1986 (amended 2012) in UK animal facilities (i.e. Named Animal Care & Welfare Officer, Named Veterinary Surgeon). Participants were provided with study instructions, including images of mice with fight wounds, to help identify aggression-related injuries. Participants were also invited to view an online video tutorial providing step-by-step instructions on how to collect and submit data to the study www.nc3rs.org.uk/laboratory-mouse-aggression-study.

Technicians were asked to observe group-housed male mice during daily routine cage checks and to record information on incidents of aggression over a consecutive four-week period (between 1 September and 30 November 2017). Technicians completed an Excel template (see Appendix) which was available to download from the NC3Rs website. The template consisted of the following:

- **A1 Consent** – Completed by the Facility Manager to provide institutional consent for participating in the study. Technicians also provided their individual consent to participate.
- **A2 Standard conditions** – Questionnaire focused on the standard husbandry conditions across the facility. Participants responded to the questions using a dropdown list of answers to aid data gathering and standardisation.
- **A3 Injury log** - Information gathered for each incident of aggression-related injury including:
  - The date of the incident, the identification number of the injured mouse, type of cage, the number of mice housed together in the cage and if the mice were in an experimental study or awaiting use (stock animals).
  - Background information on the mouse strain, including wild-type or genetically altered. Details of the specific genetic modification were not required.
  - For mice sourced from an external supplier, further information was requested about the mice in the shipment.
  - For mice in an experimental study, information about the date of the last procedure, the procedure undertaken, total number of procedures and if all mice in the cage were subjected to the same type and number of procedures.
  - Details of the aggression-related injuries (location of hair loss or wounds, such as bites and scratches) and any behavioural disturbances.
  - Actions taken to minimise further injury to the mice (e.g. removing bully mouse or adding cage enrichment).
  - Observations over a seven-day period following the date of injury to note whether the actions taken to minimise further injury to the cage were effective.
- **A4 Total number of mice –** Total number of male mice and cages of each strain housed in the facility over the data collection period. Participants were provided with further guidance and a table to help calculate the total number of mice and cages, taking into consideration mice received and culled during the data collection period. See Supplementary File 2 for guidance provided to participants to calculate total mice numbers.

All data submitted to the NC3Rs was treated as confidential, anonymised and held securely according to a Data Management Plan. Following data submission, the NC3Rs carried out quality checks of the data provided and queried facilities to ensure a full and complete data set was obtained. Common queries included checking all relevant background information was provided; clarifying if the seven-day follow up period was completed; ensuring data was collected during a defined data collection period; and ensuring only one injured mouse was documented in each row of the spreadsheet. Data was excluded from males housed with females (i.e. breeding colonies).

### Participating facilities

In total, 44 facilities participated from nine countries (Canada (n = 1), Denmark (n = 1), Germany (n = 1), Poland (n = 1), The Netherlands (n = 1), Sweden (n = 1), Switzerland (n = 1), USA (n = 2) and the UK (n = 35)). The type of facilities that participated in the study included universities, contract research organisations, biotech and pharmaceutical companies, government and charitable facilities. A total of 143 animal technicians participated in the study. 120 animal technicians based in the UK were each awarded 10 Continuing Professional Development (CPD) points from the UK IAT for participating in the study.

### Statistical analysis

Data from each facility were collated into a master Excel spreadsheet (see Supplementary File 1) and were analysed using MLwiN Version 3.00^47^. Prevalence of aggression in different mouse strains was calculated and compared to investigate differences using a chi-squared independence test (the simplest appropriate test for comparing counts of aggressive incidents for each strain). Multilevel logistic regression models were used to test hypotheses regarding the relationship between aggression occurring in the presence of a standard condition (i.e. number of mice in cage, cage cleaning protocol) whilst accounting for heterogeneity within facilities. Odds ratios were calculated via the coefficients of the multilevel logistic regression models to provide an estimate of the odds of aggression occurring in the presence of a standard condition variable versus its absence. For example, we give the odds of aggression for keeping three mice per cage versus any other number of mice per cage rather than choosing a base category such as two mice per cage.

## Supplementary information

Dataset 1
