# Supplementary material for: Cage aggression in group-housed laboratory male mice: an international data crowdsourcing project
Source: Sci Rep. 2019 Oct 23;9:15211. doi: 10.1038/s41598-019-51674-z (PMC6811576; doi:10.1038/s41598-019-51674-z)
Supplement: Supplementary file 1 — Dataset 1 [file 41598_2019_51674_MOESM1_ESM.docx]

**Cage aggression in group-housed laboratory male mice: an international data crowdsourcing project**

Katie Lidster*^1^, Kathryn Owen^1^, William J. Browne^2^, Mark J. Prescott^1^

^1^National Centre for the Replacement, Refinement and Reduction of Animals in Research (NC3Rs), Gibbs Building, 215 Euston Road, London, NW1 2BE, UK

^2^School of Education, University of Bristol, 35 Berkeley Square, Bristol, BS8 1JA, UK

Supplementary data

## Supplementary File 1

Template spreadsheet for collecting data – available [here](https://www.nc3rs.org.uk/laboratory-mouse-aggression-study)

## Supplementary File 2

Instructions for completing mouse aggression study – available [here](https://www.nc3rs.org.uk/sites/default/files/Instructions%20for%20completing%20mouse%20aggression%20study%20data%20collection-1.pdf)

## Supplementary Table 1

Number of aggression-related injuries and cages of mice with aggression-related injuries for 40/44 facilities participating in the study used to calculate the prevalence of aggression (grey = UK facilities).

| **Facility** | **Mice** | | | **Cages** | | |
| --- | --- | --- | --- | --- | --- | --- |
|  | **Number of mice with aggression-related injuries** | **Total number of mice** | **Prevalence of aggression-related injuries** | **Prevalence of aggression-related injuries** | **Number of cages with aggression-related injuries** | **Total number of cages with aggression-related injuries** |
| 1 | 114 | 13592 | 0.0084 | 92 | 2740 | 0.0336 |
| 2 | 6 | 350 | 0.0171 | 6 | 106 | 0.0566 |
| 3 | 0 | 329 | 0.0000 | 0 | 82 | 0.0000 |
| 4 | 10 | 6714 | 0.0015 | 5 | 2242 | 0.0022 |
| 5 | 62 | 920 | 0.0674 | 27 | 295 | 0.0915 |
| 6 | 47 | 507 | 0.0927 | 15 | 193 | 0.0777 |
| 7 | 7 | 2124 | 0.0033 | 5 | 657 | 0.0076 |
| 8 | 8 | 1554 | 0.0051 | 2 | 322 | 0.0062 |
| 9 | 13 | 760 | 0.0171 | 10 | 186 | 0.0538 |
| 10 | 5 | 408 | 0.0123 | 1 | 64 | 0.0156 |
| 11 | 38 | 3258 | 0.0117 | 23 | 906 | 0.0254 |
| 12 | 13 | 3738 | 0.0035 | 11 | 1604 | 0.0069 |
| 13 | 15 | 3236 | 0.0046 | 8 | 1085 | 0.0074 |
| 14 | 19 | 309 | 0.0615 | 15 | 109 | 0.1376 |
| 15 | 1 | 1445 | 0.0007 | 1 | 470 | 0.0021 |
| 16 | 18 | 10720 | 0.0017 | 6 | 6213 | 0.0010 |
| 17 | 17 | 1129 | 0.0151 | 3 | 177 | 0.0169 |
| 18 | 22 | 11200 | 0.0020 | 16 | 5170 | 0.0031 |
| 19 | 26 | 1429 | 0.0182 | 8 | 398 | 0.0201 |
| 20 | 8 | 213 | 0.0376 | 7 | 51 | 0.1373 |
| 21 | 42 | 27206 | 0.0015 | 80 | 11965 | 0.0067 |
| 22 | 5 | 634 | 0.0079 | 5 | 270 | 0.0185 |
| 23 | 8 | 260 | 0.0308 | 3 | 92 | 0.0326 |
| 24 | 21 | 1543 | 0.0136 | 8 | 655 | 0.0122 |
| 25 | 7 | 250 | 0.0280 | 3 | 60 | 0.0500 |
| 26 | 0 | 3240 | 0.0000 | 0 | 1250 | 0.0000 |
| 27 | 0 | 80 | 0.0000 | 0 | 18 | 0.0000 |
| 28 | 4 | 151 | 0.0265 | 5 | 45 | 0.1111 |
| 29 | 27 | 1012 | 0.0267 | 24 | 749 | 0.0320 |
| 30 | 4 | 81 | 0.0494 | 4 | 34 | 0.1176 |
| 31 | 28 | 7301 | 0.0038 | 5 | 649 | 0.0077 |
| 32 | 16 | 8587 | 0.0019 | 16 | 698 | 0.0229 |
| 33 | 9 | 1229 | 0.0073 | 3 | 253 | 0.0119 |
| 34 | 49 | 2433 | 0.0201 | 9 | 440 | 0.0205 |
| 35 | 0 | 1285 | 0.0000 | 0 | 424 | 0.0000 |
| 36 | 0 | 2825 | 0.0000 | 0 | 808 | 0.0000 |
| 37 | 24 | 5110 | 0.0047 | 9 | 1367 | 0.0066 |
| 38 | 0 | 6348 | 0.0000 | 0 | 1758 | 0.0000 |
| 39 | 1 | 538 | 0.0019 | 1 | 140 | 0.0071 |
| 40 | 25 | 3532 | 0.0071 | 10 | 667 | 0.0150 |
|  | Mean prevalence = 0.0153 | | | Mean prevalence = 0.0294 | | |

## Supplementary Table 2

Number of aggression-related injuries and cages of mice with aggression-related injuries from the top 10 most commonly reported strains.

| **Strain** | **Mice** | | | **Cages** | | |
| --- | --- | --- | --- | --- | --- | --- |
|  | **Number injuries** | **Total number of mice** | **Prevalence of aggression** | **Number of cages with injuries** | **Total number of cages** | **Prevalence of aggression** |
| 129S | 10 | 5,113 | 0.00196 | 10 | 1,307 | 0.00765 |
| C57BL/6 | 411 | 78,487 | 0.00524 | 247 | 26,613 | 0.00928 |
| BALB/C | 46 | 6,292 | 0.00731 | 11 | 697 | 0.01578 |
| FVB | 59 | 4,388 | 0.01345 | 27 | 1,114 | 0.02424 |
| CD1 | 52 | 2,443 | 0.02129 | 32 | 680 | 0.04706 |
| DBA | 12 | 509 | 0.02358 | 8 | 92 | 0.08696 |
| CBA | 29 | 937 | 0.03095 | *Data not available* | | |
| C3H | 11 | 257 | 0.04280 | 7 | 74 | 0.09460 |

## Supplementary Table 3

Standard husbandry practices at participating facilities (n=40 facilities). Multiple answers to questions were possible.

| **Standard husbandry practice** | | **Number of facilities** |
| --- | --- | --- |
| Are male and female mice housed in the same room? | Yes | 38 |
|  | No | 2 |
| Standard method of identification | Ear notch | 30 |
|  | Microchip | 3 |
|  | Marker pen | 3 |
|  | Toe clipping | 2 |
|  | Ear tag | 2 |
|  | Visual inspection | 1 |
|  | Other | 1 |
|  | Tail tattoo | 0 |
|  | None | 2 |
| Standard number of male mice housed per cage | 3/cage | 6 |
|  | 4/cage | 9 |
|  | 5/cage | 17 |
|  | 6/cage | 3 |
|  | 7/cage | 1 |
|  | 8/cage | 3 |
|  | 9/cage | 0 |
|  | 10/cage | 1 |
| How are mice selected into cage? | Littermates | 27 |
|  | Age | 13 |
|  | Genotype | 6 |
|  | Randomly | 5 |
|  | Mating | 2 |
|  | Weight | 1 |
|  | Other | 3 |
| Routine supplier(s) | In-house | 21 |
|  | Supplier A | 28 |
|  | Supplier B | 17 |
|  | Supplier C | 12 |
|  | Supplier D | 5 |
|  | Supplier E | 2 |
|  | Supplier F | 0 |
|  | Other | 1 |
| Age at weaning | 18 days | 3 |
|  | 19 days | 3 |
|  | 20 days | 4 |
|  | 21 days | 37 |
|  | 22 days | 1 |
|  | 28 days | 0 |
| Routine method of handling | Tail | 25 |
|  | Tail and refined methods | 5 |
| Frequency of handling of mice awaiting use (stock mice) | Daily | 3 |
|  | Weekly | 35 |
|  | Other | 2 |
| Frequency of handling of experimental mice | Daily | 10 |
|  | Weekly | 18 |
|  | Other | 11 |
| Bedding material in the cage | Aspen bedding | 24 |
|  | Dust free shavings | 7 |
|  | Corn cob | 3 |
|  | Pure-o'Cel | 1 |
|  | None | 0 |
|  | Other | 5 |
| Nesting material in the cage | Sizzle nest/Z-nest | 14 |
|  | Shred-bed/paper | 11 |
|  | Nestlet | 10 |
|  | Happi mat | 2 |
|  | Cocoon | 1 |
|  | Hay | 0 |
|  | Other | 11 |
|  | None | 0 |
| Standard cage enrichment | Chew block/wool sticks | 25 |
|  | Cardboard tunnel | 23 |
|  | House/shelter (red plastic) | 13 |
|  | House/shelter (cardboard) | 12 |
|  | Polycarbonate tunnel | 4 |
|  | Ladders | 0 |
|  | Hammock | 0 |
|  | Running wheel | 0 |
|  | Other | 7 |
|  | None | 0 |
| Cage cleaning protocol | Spot change as needed | 13 |
|  | Full cage change every week | 23 |
|  | Full cage change every two weeks | 15 |
|  | Other | 2 |
| Bedding material transferred? | Yes-pinch | 17 |
|  | Yes-handful | 5 |
|  | No | 18 |
| Nesting material transferred? | Yes-pinch | 11 |
|  | Yes-handful | 13 |
|  | Yes-all | 14 |
|  | No | 6 |
| Cage type | IVC | 29 |
|  | Conventional | 18 |
|  | Filter top | 4 |
|  | Other | 0 |
| Cage manufacturer | Techniplast | 34 |
|  | North Kent Plastics | 4 |
|  | Allentown | 3 |
|  | Lab Products | 0 |
|  | Other | 2 |
| Cage model/dimensions | GM500 | 11 |
|  | 1145T | 11 |
|  | 1284L | 9 |
|  | 1291H | 4 |
|  | 1285L | 2 |
|  | 1264C | 1 |
|  | 1290D | 1 |
|  | Cage system with external bottle (500cm^2^ floor space) | 1 |
|  | 1144B | 1 |
|  | Super mouse 1800 ventilated | 0 |
|  | Super mouse 750 ventilated | 0 |
|  | Cage system with external bottle (535cm^2^ floor space) | 0 |
|  | Other | 11 |
| Number of air changes/hour (room) | >16 | 6 |
|  | 16-22 | 23 |
|  | 20 | 4 |
|  | 50 | 1 |
|  | 60 | 0 |
|  | 75 | 0 |
|  | Other | 3 |
| Number of air changes/hour (rack) | 16-22 | 4 |
|  | 20 | 2 |
|  | 50 | 4 |
|  | 60 | 4 |
|  | 75 | 18 |
|  | Other | 8 |
| Diet | RM1 (P) | 3 |
|  | RM1 (E) | 6 |
|  | RM3 (P) | 6 |
|  | RM3 (E) | 8 |
|  | CRM (P) | 3 |
|  | Labdiet 5021 | 3 |
|  | Labdiet 5053 | 2 |
|  | Other | 18 |
| Feeding frequency | Ad libitum | 39 |
|  | Restricted | 1 |
| Food placement | Hopper | 37 |
|  | Scattered through cage | 2 |
|  | Other | 1 |
| Water type | Filtered chlorinated water | 14 |
|  | Filtered autoclaved water | 7 |
|  | Other | 20 |
| Water placement | Water bottle | 32 |
|  | Automated | 6 |
|  | Other | 3 |
| Water frequency | Ad libitum | 40 |
|  | Restricted | 1 |
| Light cycle | 12:12 | 40 |
|  | Reverse 12:12 | 1 |
|  | Other | 1 |
| Room temperature (°C) | 19-23°C | 22 |
|  | 20°C | 1 |
|  | 20-24°C | 12 |
|  | 21-23°C | 1 |
|  | 21°C | 4 |
| Room humidity setting | 40-70% | 10 |
|  | 45-65% | 28 |
|  | Other | 2 |
